#  Genetic Variants in Vitamin-D Metabolism Genes (rs1155563, rs12785878 and rs10500804) among Females with Type-2 Diabetes Mellitus in Saudi Arabia

**DOI:** 10.12669/pjms.40.8.9318

**Published:** 2024-09

**Authors:** Shatha Alharazy

**Affiliations:** 1 Shatha Matoug Alharazy, MBBS, MRes, PhD Assistant Professor in Department of Physiology, Faculty of Medicine, King Abdulaziz University, Jeddah 21589, Kingdom of Saudi Arabia. Email: smalharazy@kau.edu.sa

**Keywords:** Vitamin-D genetic polymorphism, Vitamin-D metabolism genes, Type 2 Diabetes, DHCR7, CYP2R1, Vitamin-D binding protein

## Abstract

**Background & Objective:**

Hypovitaminosis D has shown to be linked with T2DM development and control in numerous studies. The association of SNPs in genes related to VitD metabolism with T2DM has not been sufficiently studied. Consequently, our aim in the present study was to explore the association between genetic variants in genes connected with VitD, mainly a SNP in GC (rs1155563), a SNP in DHCR7 (rs12785878) and a SNP in CYP2R1 (rs10500804) with glycaemic parameters in females with T2DM in Saudi Arabia.

**Methods:**

The cross-sectional study included 149 females (age 38-52 years) with T2DM from Jeddah, Saudi Arabia (September 2022-March 2023). Blood was extracted from the participants for biochemical tests including measuring VitD [25(OH)D] concentration, parameters of glycaemia (HbA1c, insulin, fasting glucose and insulin sensitivity indices including HOMA2-IR and HOMA2-%β), and for genomic DNA isolation. Sanger DNA sequencing was used to screen for VitD genetic polymorphisms (rs1155563, rs12785878 and rs10500804).

**Results:**

Minor allele frequency for rs1155563C, rs12785878T and rs10500804G was 0.21, 0.23 and 0.37, respectively. Levels of 25(OH)D and glycaemic parameters as well did not show any significant difference between the genotypes of each SNP.

**Conclusion:**

This study showed lack of association of rs1155563 in GC, rs12785878 in DHCR7 and rs10500804 in CYP2R1 with VitD level primarily and with glycaemic parameters secondarily. Additional research is required to explore further other VitD genetic polymorphisms influencing T2DM which might lead consequently to genetically-based personalized management for T2DM.

## INTRODUCTION

Several factors including environmental, nutritional, genetic facors can influence the development of Type-2 Diabetes Mellitus (T2DM) which is extremely prevalent in Saudi Arabia.^1^ Among the important nutritional factors that appeared eminently in the research field is Vitamin-D (VitD). VitD deficiency has been found to be linked with T2DM in several studies.^2^,^3^ In addition, a number of studies have also shown that insufficient VitD level can rise the susceptibility of development of T2DM and VitD supplemental treatment can decrease the risk of development of T2DM or improve the glycemic control in T2DM patients.^4^

Genetic factors can influence VitD level [which is mainly represented by level of serum 25-hydroxyvitamin-D (25(OH)D)] due to its high heritability (53%).^5^ It has been demonstrated in genome-wide association studies and candidate gene studies that single nucleotide polymorphisms (SNP) in VitD related genes have a substantial effect on 25(OH) D level.^6^ These genetic polymorphisms were evident in enzymes and proteins involved in VitD metabolic pathway including enzymes responsible for 25(OH)D activation [25-hydroxylase (CYP2R1) and 1-hydroxylase (CYP27B1)] and elimination [24-hydroxylase (CYP24A1)] and proteins for 25(OH) D transfer [VitD binding protein (GC)] and binding [vitamin-D receptor (VDR)]. In addition to 7-dehydrocholesterol reductase (DHCR7) which facilitates the transformation of 25(OH)D to cholesterol.^7^

A large number of studies have shown significant link of certain SNPs in VDR and impaired insulin secretion and sensitivity as well as increased risk of T2DM.^7^,^8^ Only a few studies investigated SNPs in GC (the main carrier of VitD) in T2DM and found that these SNPs increase the risk of T2DM.^9^ In regards with CYP2R1, only a single recent study conducted in Chinese population by Wang et al.^10^ Has found an association between two SNPs in CYP2R1 and T2DM risk. The relationship between SNPs in Vitamin-D genes and T2DM has not been sufficiently addressed. Most of the studies focused mainly on the association between SNPs in VDR and T2DM.^11^ To our knowledge, little is known about genetic polymorphisms in VDBP (rs1155563), DHCR7 (rs12785878) and CYP2R1 (rs10500804) as new genetic markers for T2DM. A potentially vital question is whether genetic polymorphisms in genes linked to VitD metabolic process will associate with T2DM and impact its control accordingly. We aimed in the current research to study the frequency of SNPs in VitD interrelated genes, mainly rs1155563 in *GC*, rs12785878 in *DHCR7* and rs10500804 in *CYP2R1* and their link with measures of glycaemia (insulin sensitivity indices, fasting insulin, blood sugar and c-peptide) in females diagnosed with T2DM in Kingdom of Saudi Arabia.

## METHODS

This study is a cross-sectional study that involved 149 females diagnosed with T2DM (age between 38 and 52 years) from Jeddah (the western area of Saudi Arabia), (September 2022-March 2023). SNPs in genes related to VitD metabolic pathway (specifically a SNP in *GC* (rs1155563), a SNP in *DHCR7* (rs12785878) and a SNP in *CYP2R1* (rs10500804) were assessed among the women in the study. The association of the genotypes of these SNPs with VitD [25(OH)D level] and parameters of glycaemia (HbA1c, insulin, fasting glucose, c-peptide and insulin sensitivity indices including HOMA2-IR and HOMA2-%β) was also investigated. Females who joined in the present study were referred to King Fahad Medical Research Centre (KFMRC), King Abdulaziz University (KAU), Jeddah, Saudi Arabia. All participants signed a printed detailed consent for partaking in this study.

### Ethical Approval:

The study followed ethical principles of Declaration of Helsinki. Ethical approval for the study was taken from the Research Ethics Committee in Unit of Biomedical Ethics, Center of Excellence in Genomic Medicine Research (CEGMR), King Abdulaziz University (KAU) (Ref No. 013-CEGMR-02-ETH; dated July 30, 2018).

### Inclusion & Exclusion Criteria:

Participants in this study were selected according to particular inclusion and exclusion criteria. Women included in this study were formerly diagnosed with T2DM based on the recommendations of the American Diabetes Association^12^ which diagnose patients with T2DM if fasting plasma glucose ≥7 mmol/L or HbA1c ≥48 mmol/mol.12 Any woman having history of kidney or liver disease, rheumatoid arthritis, malabsorption syndrome, cancer, endocrinal disorder such as hyperthyroidism, hyperparathyroidism was excluded. In addition, any woman who reported any intake of medicines that have potential influence on VitD level (such as VitD, glucocorticoids and anticonvulsants) was also excluded Participants showing serum concentrations of creatinine and liver enzymes higher than the standard medical range were ruled out from the study (serum creatinine above 105μmol/L; Aminotransferase (AST) above 45 U/L; Alanine Aminotransferase (ALT) above 50 U/L and Alkaline Phosphatase (ALP) above 280 U/L) as well as participants with low thyroid stimulating hormone (TSH) levels (below 0.465 mIU/L).

### Study process and blood biochemical tests:

Anthropometric measures were taken from all participants as well as blood samples that were taken and stored in free of additives (clot activators, anticoagulants, preservatives or separator material) tubes and tubes containing the anticoagulant ethylenediaminetetraacetic acid (EDTA). Quantification of 25(OH)D, intact PTH, insulin and c-peptide levels in serum was performed through chemiluminescence immunoassay (CLIA), with a LIAISON auto-analyzer (DiaSorin Inc., Stillwater, MN, USA). The intra-assay and inter-assay coefficient of variation (CV) for the analyzed samples were < 5%. Liver enzymes, creatinine, blood glucose, magnesium (Mg), calcium (Ca), phosphate (PO_4)_, albumin and lipid profile were analyzed in serum through the colorimetric method utilizing a VITROS 250 Clinical Chemistry auto-analyzer (Ortho-Clinical Diagnostics Inc., Rochester, NY, USA). The samples showed an intra-assay CV of 3.5% and an inter-assay CV of 3.9%. VitD deficiency was determined according to Institute of Medicine (IOM) recommendations.^13^ These guidelines define 25(OH)D concentration lower than 12 ng/ml as VitD deficiency, 25(OH)D level between 12 and 19 ng/ml as VitD insufficiency, and level from 20 to 50 ng/ml as VitD sufficiency.

Serum high sensitive C-reactive protein (hs-CRP) was analyzed through immunoassay, with a VITROS 5,1 FS chemistry auto-analyzer (Ortho-Clinical Diagnostics Inc., Rochester, NY, USA). The samples showed an intra-assay and inter-assay CV of 4.2% and 4.5% correspondingly. Serum thyroid function test (TFT) [including thyroid stimulating hormone (TSH), free triiodothyronine (T4) and free thyroxin (T3)] was analyzed by immunoassays, using VITROS ECiQ (Ortho-Clinical Diagnostics Inc., Rochester, NY, USA). Glycosylated hemoglobin (HbA1c) was quantified by a VITROS 5,1 FS chemistry auto-analyzer (Ortho-Clinical Diagnostics Inc., Rochester, NY, USA). HbA1c intra and inter-assay CV were < 4%.

Homeostasis Model Assessment 2 (HOMA2) was calculated in this study to evaluate insulin resistance (HOMA2-IR) and β-cell function (HOMA2-%β). HOMA2-IR and HOMA2-%β were estimated in a constant status from fasting glucose (3-25 mmol/L), fasting insulin (2.88–43.16 mIU/L) and fasting c-peptide (0.6-10.5 μU/ml) with the aid of a PC HOMA calculator program (version 2.2.3) supplied by University of Oxford Diabetes Trials Unit, accessible at https://www.dtu.ox.ac.uk/homacalculator/. Fasting insulin and HOMA2 were not measured in women who were taking insulin as intake of exogenous insulin can affect these parameters.

### Genetic polymorphisms screening:

Genomic DNA was isolated initially using DNA extraction kit (53104, Qiagen, Hilden, Germany). Assessment of the concentration and purity the DNA filtrate was done using NanoDrop spectrophotometer (ND-1000 UV-VIS). Screening for specific SNPs in genes related to VitD metabolism (rs1155563 in *GC*, rs12785878 in *DHCR7* and rs10500804 in *CYP2R1*) was conducted. First, primers’ designs for these SNPs were prepared with web-based Primer3 (v. 0.4.1) program (Table-I). PCR purification kit then was used to amplify and purify DNA samples. Finally, Sanger sequencing was performed through genetic analyzer (3500 genetic analyzer, Applied Biosystems, Thermo Fisher Scientific, Waltham, MA, USA) and BigDye Terminator V3.1 Cycle Sequencing kit (cat#4337455, Applied Biosystems, ThermoFisher Scientific, MA, USA).

**Table-I:** The design of PCR primers used for the selected SNPs in GC, DHR7 and CYP2R1 genes.

GENE	Primer Design	PCR size (bp)	*PCR (°C)
GC	F: 5′- TTACATTCCAATTGCCACCA -3′ R: 5′- CCCATCAACCCACCATCTAC -3′	480	60
DHCR7	F: 5′- CGTTGTGGGATCTGGAAGTT -3′ R: 5′- CAGCAGACAGGACATGAGGA -3′	455	
CYP2R1	F: 5′- CTGCTTTGAACCACACATGG -3′ R: 5′- ACACCCGCCTTTGTGTTAGT -3′	532	

* Annealing PCR temperature. F is forward and R is reverse.

Normally distributed data are presented as mean±SD. Non-normally distributed data are presented as median (IQR). Descriptive data are presented as n (%). (%) is percentage out of the total number of subjects. BMI is Body Mass Index; 25(OH)D is 25-hydroxyvitamin-D; OHD is oral hypoglycemic drug; PTH is parathyroid hormone; Ca is calcium; PO4 is phosphate; Mg is magnesium; HDL-C is high density lipoprotein cholesterol; LDL-C is low density lipoprotein cholesterol; VLDL-C is very low density lipoprotein cholesterol; Hs-CRP is high sensitivity C-reactive protein. AST is Aspartate Aminotransferase; ALT is Alanine Aminotransferase; ALP is Alkaline Phosphatase; TSH is thyroid stimulating hormone; free T4 is free thyroxin; and T3 is free triiodothyronine. HOMA2-IR is homeostatic assessment 2 for insulin resistance. HOMA2-%β is homeostatic assessment 2 for β-cell function; HOMA2-IR/% β C-peptide was calculated using fasting glucose and C-peptide; HOMA2- IR/% β insulin was calculated using fasting glucose and fasting insulin. *Fasting insulin, HOMA2-IR and -%β insulin were measured only in subjects not taking insulin (n=87).

### Statistical analysis:

It was done by application of SPSS program (v.20 SPSS Chicago Inc, 2011). Kolmogorov-Smirnov test was applied to test for data normality. Descriptive data were depicted as a percent of the whole samples number. Numerical parametric and non-parametric data were demonstrated as means ± SD and median (IQR), respectively. For comparison between genotype groups of each SNP, Kruskal-Wallis H test was applied due the skewed distribution of data. Results with P value ≤ 0.05 were regarded as statistically significant. Fully informed, written consent was obtained from the participants.

## RESULTS

General and biochemical results of the included females are shown in Table-II. Median of VitD [25(OH) D] level in the participating females with T2DM was 12 ng/ml. Based on IOM^21^ VitD status guidelines, 50% had VitD deficiency, 32% had VitD insufficiency, and 18% had optimal VitD levels.

**Table-II:** General and biochemical characteristics of the participating women.

Variable	Results (n=149)
Age (years)	46 (38-52)
Years since T2DM diagnosis (years)	5 (2-15)
***T2DM treatment***	
Diet	2 (1%)
OHD	79 (53%)
Diet + OHD	6 (4%)
Insulin	12 (8%)
Insulin+OHD	50 (34%)
BMI (kg/m²)	35 ± 8
Serum 25(OH)D (ng/ml)	12 (8-18)
Serum Intact PTH (pg/ml)	45.6 (33.5-64)
Serum Albumin (g/L)	44 (40-48)
Serum Ca (mmol/L)	2.2± 0.23
Serum PO_4_ (mmol/L)	1.16 (1.07-1.27)
Serum Mg (mmol/L)	0.7 (0.6-0.7)
Serum total cholesterol (mmol/L)	4 (3.4-4.6)
Serum triglyceride (mmol/L)	1.41 (1.05-2.4)
Serum HDL-C (mmol/L)	1.1 (0.8-1.2)
Serum LDL-C (mmol/L)	2.05 (1.75-2.91)
Serum VLDL-C (mmol/L)	0.63 (0.48-1.11)
Serum AST (U/L)	16 (11-22)
Serum ALT (U/L)	30 (27-34)
Serum ALP (U/L)	69 (45-93)
Serum creatinine (µmol/L)	46 (38-59)
Serum hs-CRP (mg/L)	6.6 (2.9-13)
Serum TSH (mIU/L)	2.13 (1.40-3.01)
Serum Free T_4_ (pmol/L)	16.5±3.03
Serum Free T_3_ (pg/ml)	3.8 (3.4-4.2)
Fasting insulin (pmol/L)*	10.5±5.3
Fasting c-peptide (nmol/L)	3.3±1.1
Fasting glucose (mmol/L)	7.5 (5.8-10.8)
HbA1c (mmol/mol)	66±7
HOMA2-IR insulin*	1.7 (0.84-2.4)
HOMA2-%β insulin*	59 (28-94)
HOMA2-IR C-peptide	2.7 (1.6-3.6)
HOMA2-%β C-peptide	75 (42-112)

**Fig.1:**
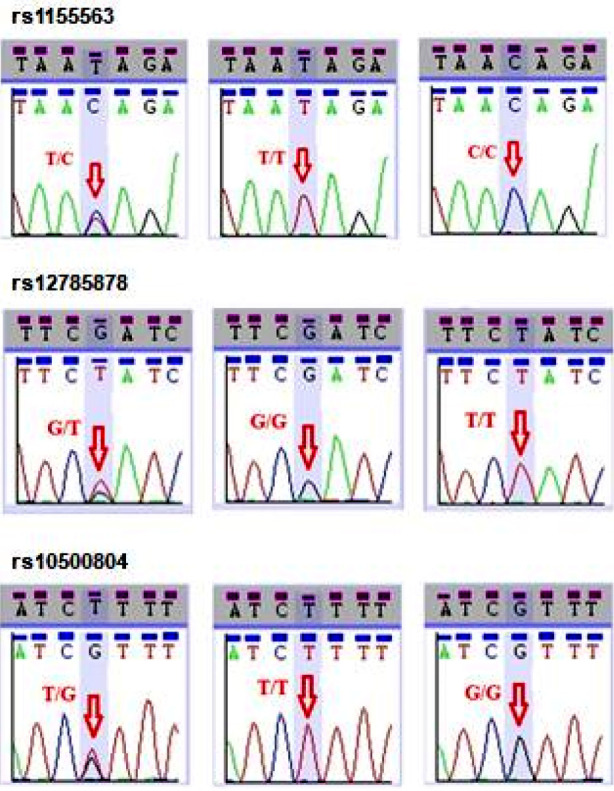
Chromatograms obtained from Sanger sequencing chromatograms representing rs1155563, rs12785878 and rs10500804 in the studied women with T2DM (n=149).

**Fig.2:**
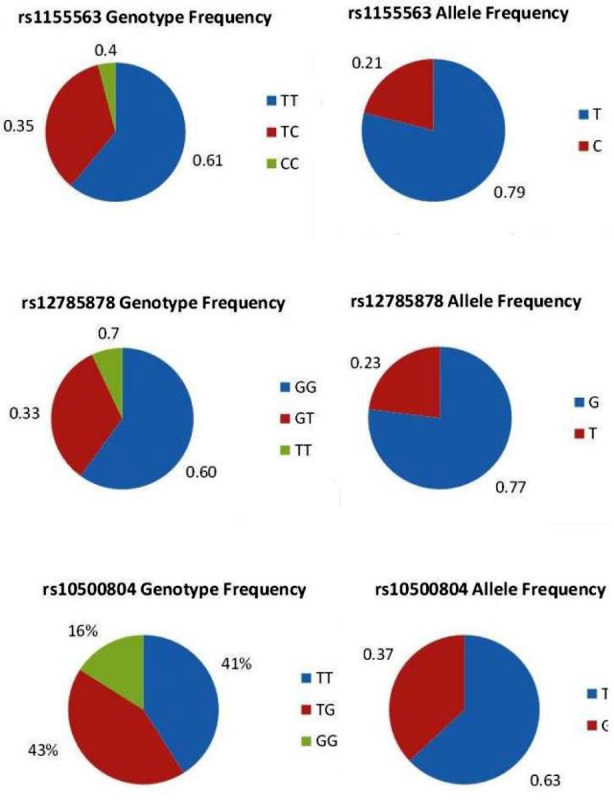
Frequencies of genotypes and alleles of rs1155563, rs12785878 and rs10500804 in the studied women with T2DM (n=149).

Sanger sequencing chromatograms showed the different variants of the studied SNPs (Fig.1). The genotypes and alleles frequencies of rs1155563, rs12785878 and rs10500804 are presented in Fig.2. Majority of the participants (around 60%) had the reference genotype TT and GG for rs1155563 and rs12785878, respectively. For rs10500804, 43% of the women had heterozygous TG and 41% had the reference genotype TT. It was also observed that minor allele frequency (MAF) for rs1155563C was 0.21.

In addition, MAF for rs12785878T and rs10500804G was 0.23 and 0.37, respectively. VitD level and levels of glyaemic parameters among the subcategorized genotypes of rs1155563, rs12785878 and rs10500804 are shown in Table-III. The results showed no significant difference in levels of 25(OH)D, HbA1c, fasting glucose, fasting insulin, fasting C-peptide and insulin sensitivity indices including (HOMA2-IR and HOMA2-%β) between the genotypes of each SNP. In addition, there was no significant difference in bone parameters, lipid profile, LFT, creatinine and TFT between the three genotypes of the studies SNPs.

**Table-III:** Vitamin-D and glycaemic parameters levels in the different genotypes of the studied SNPs.

Variable (N= 149)	rs1155563 (GC)			P-value	rs12785878 (DHCR7)			P- value	rs10500804 (CYP2R1)			P-value
		
	TT	TC	CC		GG	GT	TT		TT	TG	GG	
Serum 25(OH)D (ng/ml)	13 (8-18)	9 (8-16)	15 (8-16)	>0.05	11 (8-16)	13 (8-18)	14 (9-17)	>0.05	10 (7-16)	14 (10-19)	9 (8-15)	>0.05
Fasting glucose (mmol/L)	7.9 (5.9-10.1)	6.5 (5.3-12.5)	6.6 (6.1-7)		7.9 (6.6-11.3)	6.1 (5.2-8.4)	8.9 (4.2-10.6)		7.3 (5.8-9.9)	6.9 (5.5-10.6)	10.6 (7.3-13.5)	
Fasting insulin (pmol/L)*	11.7 (6.9-19.8)	10.5 (5.9-15.5)	4.2 (3.7-4)		11.5 (4.9-15.2)	11.4 (6.8-21)	7.3 (6.4-8)		7.3 (6.4-8.4)	11.4 (5.5-20.7)	12.6 (7.7-19.6)	
Fasting c-peptide (nmol/L)	2.7 (1.9-4.1)	2.8 (1.5-4.3)	1.8 (1.7-1.9)		2.7 (1.9-4.4)	2.7 (1.3-3.9)	2.5 (2.4-3)		2.4 (1.4-3.9)	2.5 (1.6-3.9)	3.5 (2.3-4.7)	
HbA1c (mmol/mol)	63 (51-78)	61 (46-91)	62 (60-64)		64 (51-83)	61 (52-75)	67 (41-97		73 (41-99)	60 (51-79)	75 (55-89)	
HOMA2-IR insulin*	1.6 (1.03-2.06)	1.96 (0.79-2.77)	0.58 (0.51-0.60)		1.7 (0.96-2.7)	1.12 (0.68-2.02)	1.7 (1.1-2.2)		1.3 (0.77-1.82)	1.5 (0.72-2.5)	1.9 (1.6-3.06)	
HOMA2-%β insulin*	60 (36-101)	55 (6-94)	35 (33-36)		55.2 (26-94)	68 (44-99)	40 (11-62)		56 (30-78)	74 (9-107)	47 (22-99)	
HOMA2-IR C-peptide	2.6 (1.8-3.7)	2.8 (1.6-4.2)	1.4 (1.3-1.4)		2.6 (1.7-3.6)	2.8 (1.3-3.6)	4.07 (2.04-4.1)		2.7 (1.4-3.6)	2.1 (1.6-3.4)	3.4 (2.3-5.5)	
HOMA2-%β C-peptide	81 (35-109)	72 (43-148)	68 (62-68)		72 (37-123)	80 (47-119)	64 (15-76)		75 (43-132)	80 (35-114)	65 (43-100)	

Median (IQR) differences in 25(OH)D between SNPs categories were determined using Kruskal-wallis test. 25(OH)D is 25-hydroxyvitamin-D. HOMA2-%β is homeostatic assessment 2 for β-cell function; HOMA2-IR/% β C-peptide was calculated using fasting glucose and C-peptide; HOMA2- IR/% β insulin was calculated using fasting glucose and fasting insulin.

* Fasting insulin, HOMA2-IR and -%β insulin were measured only in subjects not taking insulin (n=87).

## DISCUSSION

The current research described the allele frequency of three SNPs in genes engaged in VitD metabolic pathway in T2DM female population in Saudi Arabia. The MAF of the SNP rs1155563 in *GC* (the gene encoding VitD carrier protein), the SNP rs12785878 in *DHCR7* (the gene encoding the enzyme needed for skin ViD synthesis) and the rs10500804 in *CYP2R1* (the gene encoding the enzyme required for VitD activation in the liver) were all described in this study. The MAF of rs1155563 T/C was 0.21 which was identical to MAF seen in another study in Arabs, while MAF of rs10500804 T/G was 0.37 in comparison to a MAF of 0.42 seen in the same Arab study.^14^

On the other hand, MAF of rs12785878 G/T observed in Saudi Arabian population was hugely different (0.23) than the MAF reported in Arabs (0.45).^14^ This inconsistency between the MAF of the two studied SNPs and the reported MAF of these SNP in the other study might be due to heterogeneous Arab ethnicities included in the other study and the inclusion of both genders compared to our study that included only women from Saudi Arabian region only.

Vitamin D deficiency has been consistently evident in subjects with T2DM. It has been linked with T2DM in several studies.^2^,^3^ Its prevalence has been reported previously to be high among patients with T2DM in these former studies. It was not surprising that we have found only 18% of the participants having optimal 25(OH)D level.

When we investigated in this study the SNPs in genes related to VitD metabolism (including rs1155563, rs12785878 and rs10500804), we did not find any association between these SNPs and glycaemic parameters. A possible explanation of this lack of association is that no association was observed between these SNPs and VitD originally, although these SNPs have been reported to have an association with VitD level.^14^ Our finding contradicts to what was found in a large study in population from Arabic origin which showed that VitD was associated with same SNPs in *GC* and *CYP2R1*.

However, the same study showed a parallel finding in respect with lack of association of VitD with rs12785878 (the studied SNP in *DHCR7*).^14^ It has been proposed by a former study that genetic variation effect on VitD level might be more evident in men compared with women.^5^ This might elucidate the absence of relationship between SNPs in VitD related genes and VitD level in this female study. Our result weakens the possibility of the influence of the studied SNPs on VitD and consequently, T2DM. This question whether genetic variation in genes involved in VitD metabolism (specifically *GC* and *CYP2R1* and *DHCR7*) can influence T2DM control. In order to confirm or disconfirm this information, larger studies are needed with perhaps including population from multiple ethnicities. As lack of association between rs1155563, rs12785878 and rs10500804 was observed only in female population of Saudi Arabia. These results might differ in other population from other ethnic backgrounds as ethnicity has a possible impact on the association of VitD with genetic variation.^11^ Moreover, including more number of SNPs in the genes *GC* and *CYP2R1* and *DHCR7* in future studies when investigating the association between VitD genes and T2DM might give a stronger support whether these VitD related genes have a vital role and involvement in T2DM.

### Limitations:

It includes the limited number of the investigated SNPs and the uncontrolled confounding factors influencing vitD level (e.g. sunlight exposure, age and obesity) as well as variations in diabetic treatment and T2D duration that might contribute to the relationship between VitD and glycemic parameters.

## CONCLUSION

This study did not discover any significant association of rs1155563 in *GC*, rs12785878 in *DHCR7* and rs10500804 in *CYP2R1* neither with VitD concentration nor with the glycaemic parameters in women with T2DM in Saudi Arabia. Further research is needed to investigate other VitD genetic polymorphisms influencing T2DM which might give a novel perception in control of T2DM based on genetic basis.
